# Research on Kinematic Calibration and Trajectory Tracking of the Dual-Robot Collaborative Grinding and Polishing System

**DOI:** 10.3390/s25134075

**Published:** 2025-06-30

**Authors:** Wenduan Yan, Luwei Xu, Yifang Sun, Hongjie Xu, Zhifei Ji

**Affiliations:** 1 School of Optoelectronic and Mechanical Engineering, Minnan University of Science and Technology, Quanzhou 362700, China; 15960246076@163.com (L.X.); sunyifang18@mails.ucas.ac.cn (Y.S.); 2 School of Advanced Manufacturing, Fuzhou University, Quanzhou 362551, China; 3 Shishi Huixing Machinery Co., Ltd., Quanzhou 362700, China; 15980858022@163.com; 4 College of Marine Equipment and Mechanical Engineering, Jimei University, Xiamen 361021, China; 15710671591@163.com

**Keywords:** dual robots, MATLAB simulation, calibration, cooperative relative motion

## Abstract

**Highlights:**

**What are the main findings?**

- Proposes a systematic solution for motion planning in dual-robot collaborative grinding and polishing systems, experimentally validating its effectiveness.
- Integrates the “handshake” method with the seven-point calibration approach via a dual-robot pose constraint model, boosting spatial mapping accuracy.

**What is the implication of the main finding?**

- Offers a reliable solution for industrial dual-robot collaborative grinding and polishing tasks, improving operation accuracy and efficiency.
- Provides a reference for kinematic modeling and calibration of other multi-robot systems, promoting the development of robotic collaborative technologies.

**Abstract:**

This study proposes a systematic solution to the motion planning challenges in dual-robot collaborative grinding and polishing systems, with its effectiveness experimentally validated. By establishing a dual-robot pose constraint model, this study innovatively integrates the “handshake” method with the seven-point calibration approach, achieving enhanced spatial mapping accuracy between the base coordinate system and tool coordinate system. Based on the modified Denavit–Hartenberg (DH) method, this study establishes kinematic modeling for EPSON C4-A901S robots on the MATLAB platform. By integrating calibration parameters, a dual-robot collaborative grinding model is constructed, with its reliability thoroughly verified through comprehensive simulations. An experimental platform integrating dual EPSON C4-series robots with grinding devices, clamping fixtures, and drive systems was established. The average error below 8 mm from 10 repeated experiments fully validates the accuracy and practical applicability of the integrated calibration method.

## 1. Introduction

With the rapid development of modern manufacturing technology, three-dimensional curved surface workpieces have been increasingly and widely applied in such fields as automobiles, aerospace, additive manufacturing, and abrasive tool manufacturing. As a key link to achieve high-precision surface processing, grinding and polishing technology have put forward higher requirements for production efficiency and product quality. For a long time, in the majority of situations, this type of work has relied on manual operation. However, owing to the adverse working environment, the high-intensity workload, and the escalating precision requirements, not only has it led to substantial labor costs, but quality problems have also frequently arisen. Consequently, it is an inescapable trend that automated [1,2,3] grinding technology is progressively replacing traditional manual work.

In the current domain of grinding and polishing, the application of robots predominantly centers around single-robot grinding [4,5]. Nevertheless, as industrial products are becoming more and more complex and diversified, and the forms of complex machined parts are constantly changing, the single-robot grinding system encounters many challenges with respect to the grinding coverage rate. Owing to the constraints on its motion and operation, it is arduous to comprehensively and efficiently cover all the sections of complex workpieces requiring grinding. The dual-robot collaborative technology offers a novel solution to this problem. In this technical solution, the system employs a dual-robot collaboration model. The master robot secures the workpiece to be processed via the flange, while the slave robot holds the grinding tool through the flange and executes the grinding and polishing operations. Specifically, the robot tasked with fixing the workpiece possesses the kinematic ability of six degrees of freedom. It is capable of dynamically adjusting its pose during the grinding operation in accordance with the demands of diverse grinding tasks and the intricate shapes of the workpieces, thereby attaining a higher level of precision and flexibility. This design is capable of ensuring that the grinding area is comprehensively covered, effectively resolving the technical problem that a single robot usually has difficulties in achieving full coverage when handling workpieces with complex shapes. Currently, in comparison to the research accomplishments in numerous fields both domestically and internationally, studies regarding the collaborative motion of dual robots [6,7,8,9] and the calibration of dual robots remain relatively scarce. Yu et al. [10] proposed a circular ring visual marker based on cross-ratio invariance, which achieves 6-DoF pose estimation through Union-Find algorithm-based edge segmentation and ellipse fitting. Within a 2–4 m working range, the system demonstrates maximum mean translation errors of 2.19–9.44 mm and rotation errors of 0.782°–1.703°, yielding 2× higher accuracy than conventional methods like AprilTag, along with superior robustness in high-illumination environments. Qin et al. [11] proposed a calibration method combining dual quaternion closed-form solutions with an iterative Levenberg–Marquardt (LM) algorithm-based approach for simultaneously calibrating hand–eye, flange–tool, and inter-robot relationships in dual-robot collaborative systems. Hua et al. [12] proposed a hand–eye calibration algorithm integrating convex relaxation-based global optimization with dual quaternions, which enhances robotic hand–eye coordination accuracy to meet the high-precision positioning requirements for switchgear operations. Wang et al. [13] addressed the kinematic calibration problem of robotic hand–eye systems by proposing a separable calibration framework based on dual quaternions. Through theoretical derivation and experimental validation, they conducted an in-depth analysis of the comparative advantages, limitations, and applicable conditions between simultaneous and separable calibration methods. Although the dual-quaternion calibration method exhibits theoretical advantages in rotational accuracy and translational accuracy based on dual quaternion theory, its industrial implementation faces dual contradictions: On one hand, the method incurs prohibitively high hardware costs, requiring CMOS industrial cameras (~USD 5000), high-precision checkerboard calibration targets (USD 200), and laser tracker systems (USD 100,000–USD 200,000), making it financially inaccessible for small- and medium-sized enterprises; on the other hand, its reliance on advanced mathematical theories including dual algebra and Lie group homomorphism theorems necessitates 3–6 months of specialized training for non-expert engineers, fundamentally conflicting with the “plug-and-play” requirements of industrial settings, particularly limiting its applicability in cost-sensitive and medium- to low-accuracy industrial scenarios.

This study proposes an integrated “Handshake + Seven-point” calibration framework based on dual-robot closed-loop kinematic constraints. The method solves base frame transformation matrices via four non-coplanar constraints of the Handshake Method [14] and refines tool frame parameters through least-squares optimization of the seven-point method [15], achieving sub-2.5 mm trajectory tracking accuracy after iterative error-decoupling optimization. Compared with dual-quaternion methods, this USD 100 solution meets the accuracy requirements for 85% industrial applications, providing SMEs with a cost-effective engineering solution with notable economic advantages. The specific implementation methodology is as follows: The EPSON C4-A901S robot is designated as the master robot for gripping the workpiece, while the EPSON C4-A601S robot is selected as the slave robot tasked with the grinding operation. Kinematic constraints are first established based on the closed kinematic chain formed by the dual-robot system. The “handshake” method and the seven-point method are then employed to precisely define the spatial transformation between the base and tool coordinate systems of the dual robots, enabling system modeling in MATLAB 2022a. Subsequently, the master and slave robots are controlled to follow predefined trajectories, with comprehensive simulation analysis conducted to validate feasibility. Finally, an experimental platform integrating EPSON C4 series robots, a grinding module, a clamping unit, and a drive system is constructed. Coordinated motion experiments are performed on the dual-robot collaborative grinding system, verifying both the reliability of the proposed approach and the accuracy of the simulation model.

## 2. Kinematic Analysis of Dual-Robot Systems

**Figure 1:**
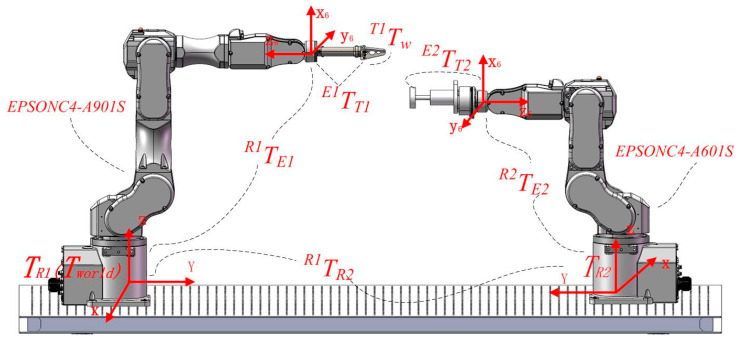
Coordinated constraint relationships in dual-robot systems.

In this study, an EPSON C4 series robotic system is employed, where the EPSON C4-A601S—selected for its high joint motion velocity and positioning accuracy—is equipped with a grinding module to function as the grinding robot, while the EPSON C4-A901 serves as the workpiece-holding robot. Figure 1 illustrates the coordinate system relationship within the dual-robot grinding and polishing system. In the figure, [R*_i_*] denotes the robot base coordinate system, [E*_i_*] represents the end-effector coordinate system of the robotic arm, and [T*_i_*] indicates the tool coordinate system of the robotic arm, where *i* = 1 corresponds to the EPSON C4-A901S robot and *i* = 2 refers to the EPSON C4-A601S robot, while [w] signifies the workpiece coordinate system. When the grinding tool establishes contact with the workpiece, the dual-robot system forms a closed kinematic chain. Based on the master–slave control strategy for system decoupling, the constraint equations of the system can be derived as follows:

(1) TwR1=TE1R1⋅TT1E1⋅TwT1

(2) TwR1=TR2R1⋅TE2R2⋅TT2E2⋅TwT1

where *^R^*^1^*T_w_* represents the transformation matrix from the workpiece coordinate system to the master robot’s base coordinate system. Upon specification of the grinding task requirements and the determination of the tool path trajectory, this transformation matrix becomes fully defined and serves as a known parameter in the system; *^E^*^1^*T_T_*_1_ and *^E^*^2^*T_T_*_2_ denote the transformation matrices from the gripping tool to the master robot and from the grinding tool to the slave robot, respectively. While both matrices are structurally defined by the mechanical assembly, *^E^*^2^*T_T_*_2_ must be calibrated owing to the unique properties of the grinding tool, resulting in a constant matrix post-calibration. The homogeneous transformation matrix *^R^*^1^*T_R_*_2_ defines the spatial relationship between the base frames of both robotic systems, which is determined through calibration procedures and remains invariant during operation. The homogeneous transformation matrix *^T^*^1^*T_w_* defines the spatial relationship between the workpiece frame and the gripping tool frame. This matrix is structurally determined by the mechanical assembly configuration, where the workpiece maintains a fixed pose relative to the gripping tool, resulting in a time-invariant transformation. The homogeneous transformation matrix *^T^*^2^*T_w_* defines the dynamic spatial relationship between the workpiece frame and the grinding device frame. As the grinding tool must maintain persistent contact with the target point throughout the process, this matrix becomes path-dependent and exhibits time-varying characteristics [16].

The equation establishes a closed-loop constraint relationship of “master robot motion—base frame transformation—slave robot motion”, rigorously maintaining time-invariant relative poses between master–slave end-effectors. This constraint equation provides the theoretical foundation for integrating the Handshake Method and the seven-point method. The Handshake Method derives the base frame transformation matrix *^R^*^1^*T_R_*_2_ to establish a global coordinate mapping for the dual-robot system, ensuring coordinated motion under a unified reference frame. The seven-point method computes the tool frame transformation matrix *^E^*^2^*T_T_*_2_ to compensate for end-effector installation errors. When orientation error Δ*R* exists in *^R^*^1^*T_R_*_2_ and position error Δ*P* occurs in *^E^*^2^*T_T_*_2_, the constraint equation transforms into (3). This constraint equation resolves the coupling between Δ*R* and Δ*P*, enabling error convergence through iterative optimization.

(3) TE1R1⋅TT1E1=TR2R1+ΔR⋅TE2R2⋅TT2E2+ΔP⋅TT1T2

### 2.1. Comparison Between Integrated Calibration Method and AprilTag Calibration Method

The integrated calibration method (“Handshake” + seven-point calibration) and AprilTag visual calibration exhibit complementary technical advantages in robotic calibration: This method relies on robotic kinematic equations and joint angle data, featuring low hardware costs and omnidirectional environmental adaptability, making it suitable for fixed-workstation calibration and basic trajectory planning of traditional industrial robots. The latter, based on visual measurement and image processing techniques, requires higher hardware costs and depends on marker visibility, yet demonstrates unique advantages in dynamic scenarios (e.g., UAV navigation andmobile robot real-time localization), high-precision fields (e.g., medical surgical robots and semiconductor manufacturing), and unstructured environments (e.g., AR/VR spatial registration), with applications spanning industrial, medical, and aerospace domains. The integrated calibration is suitable for cost-effective deployment in large-scale industrial production, while AprilTag provides core support for cutting-edge technological breakthroughs. As shown in Table 1.

### 2.2. Dual-Robot Base Frame Calibration

Kinematic analysis of the dual-robot system indicates that the calibration of *^R^*^1^*T_R_*_2_ is essential for establishing accurate inter-robot spatial relationships. Since this relative pose relationship serves as the fundamental basis for dual-robot collaborative operation, its precise measurement is imperative. The calibration of dual-robot base frames aims to determine the transformation matrix between coordinate systems R1 and R2. This study employs a calibration method in which two EPSON robots are each equipped with a calibration needle at their end-effectors. The calibration procedure involves controlling the robots to bring their needle tips into contact at four spatially distinct positions, with the critical condition that these four contact points must not lie on the same plane. Based on the coordinate relationships obtained from these non-coplanar contact points, the calibration matrix between the two robot systems is then mathematically determined. This experimental method enables direct determination of the coordinate transformation relationship between the two robotic base frames without requiring auxiliary measurement tools.

The calibration procedure is conducted as follows:

(1) Coordinate system definition and notation specification: The world coordinate system, base frames (R1 and R2 for Robot 1 and Robot 2, respectively), and tool frames (T1, T2) are explicitly defined. With calibration needles mounted on each robot’s end-effector, both manipulators are controlled to establish four non-coplanar contact points (P_1_–P_4_) within their shared workspace. The corresponding coordinates of each contact point are recorded in both robot base frames as *^R^*^1^*P_k_* and *^R^*^2^*P_k_* (k = 1, 2, 3, 4).

(2) Rotation matrix computation: Assuming the world coordinate system coincides with Robot 1’s base frame, we obtain

(4) Pbi=RR2R1⋅PR2+TR2R1

Substituting the coordinates of the four contact points into the above equations, subtracting point coordinates, and reformulating them in matrix form yields

(5) x1R1−x2R1x1R1−x3R1x1R1−x4R1y1R1−y2R1y1R1−y3R1y1R1−y4R1z1R1−z2R1z1R1−z3R1z1R1−z4R1=RR2R1⋅x1R2−x2R2x1R2−x3R2x1R2−x4R2y1R2−y2R2y1R2−y3R2y1R2−y4R2z1R2−z2R2z1R2−z3R2z1R2−z4R2

To ensure the solvability of ^*R*1^*R_R_*_2_, the selected four points must be non-coplanar (the determinant of the matrix on the right-hand side of the equation must be non-zero), from which the rotation matrix is derived as follows:

(6) RR2R1=x1R1−x2R1x1R1−x3R1x1R1−x4R1y1R1−y2R1y1R1−y3R1y1R1−y4R1z1R1−z2R1z1R1−z3R1z1R1−z4R1⋅x1R2−x2R2x1R2−x3R2x1R2−x4R2y1R2−y2R2y1R2−y3R2y1R2−y4R2z1R2−z2R2z1R2−z3R2z1R2−z4R2−1

(3) Compute the translation matrix: Substitute the rotation matrix *^R^*^1^*R_R_*_2_ into

(7) PR2R1=PRi−RR2R1⋅PR2

(4) Orthonormalization of the rotation matrix: Due to computational errors, the derived rotation matrix *^R^*^1^*R_R_*_2_ may violate orthonormality constraints. To reconcile the discrepancies between matrices while preserving geometric validity, we optimize the solution using the Frobenius norm criterion. The specific formulation is given as follows:

(8) $${\left\|A\right\|}_{F}={\left(\sum_{i=1}^{m}\sum_{j=1}^{n}{\left|a_{ij}\right|}^{2}\right)}^{1/2},\;A\in R^{m\times n}$$

Define the objective function as

(9) J=R−RR2R1F2

Constraints: $R\cdot R^{T}=I$

The optimized orthonormalized rotation matrix R∧R2R1 is obtained when the objective function J reaches its minimum.

(10) JR∧R2R1=minR⋅RT=IR−RR2R1F2

**Figure 2:**
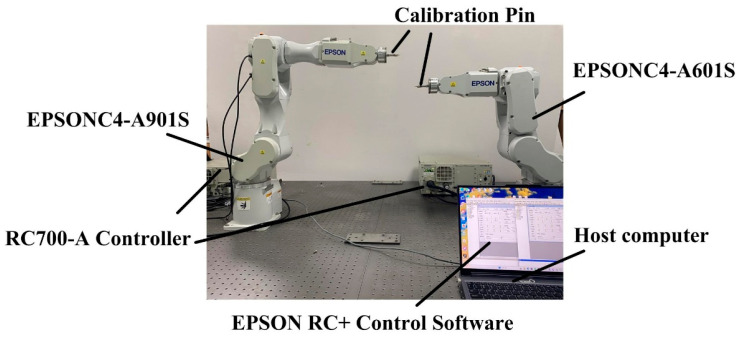
Base frame calibration experimental platform.

Base coordinate system calibration experiment:

The calibration experimental platform consists of an RC700-A controller with a host computer, two robotic manipulators, and a calibration probe, as illustrated in Figure 2. Following the experimental procedure, a dual-robot base frame calibration was conducted, with the coordinate data of key points for both the master and slave robots recorded in Table 2 and Table 3, respectively.

The rotation matrix *^R^*^1^*R_R_*_2_ is derived from Equation (6).

(11) RR2R1=1.00000.010−0.0041.0000−0.011−0.0121.006

The translation matrix *^R^*^1^*P_R_*_2_ is derived from Equation (7).

(12) PR2R1=−1085.8118.2987.362

After orthonormalization, the transformation matrix between the base frames of the two robots is obtained as

(13) TR2R1=0.99990.00390.00002−1085.81100.99990.00398.298−0.0099−0.000030.99997.3620001

### 2.3. TCP Calibration

This paper adopts the seven-point method for Tool Center Point (TCP) calibration.

**Figure 3:**
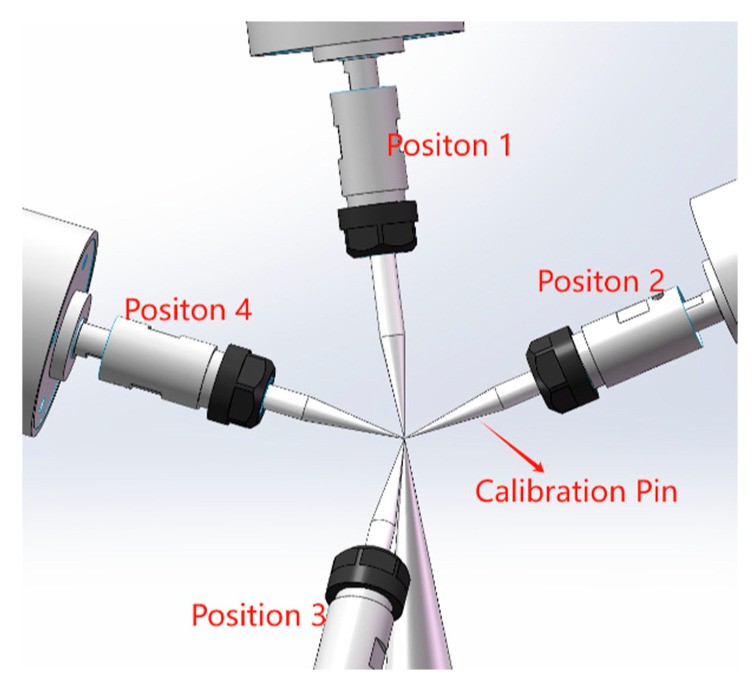
Position calibration schematic.

The calibration procedure is as follows:

(1) Determine calibration points: A calibration needle is fixed on the optical platform, with its tip position designated as P. Control the robot to position the tool end-effector at point P with four distinct orientations, as illustrated in Figure 3, while recording its pose relative to the base coordinate frame.

(2) Formulate the system of equations: Applying coordinate transformation principles yields the relevant equations. Since the tool end-effector reaches the same position four times, simultaneous elimination of point P coordinates gives the following matrix:

(14) AX=B

where matrix *A* is composed of differences between rotation matrices of the flange end coordinate system under different orientations, matrix *B* consists of corresponding positional differences, and *X* represents the Tool Center Point (TCP) position in the end-effector coordinate system (*^E^*^2^*P_T_*).

(3) Solve using the least squares method: Obtain *^E^*^2^*P_T_*_2_ by applying the least squares principle:

(15) $$X={\left(A^{T}A\right)}^{-1}A^{T}B$$

**Figure 4:**
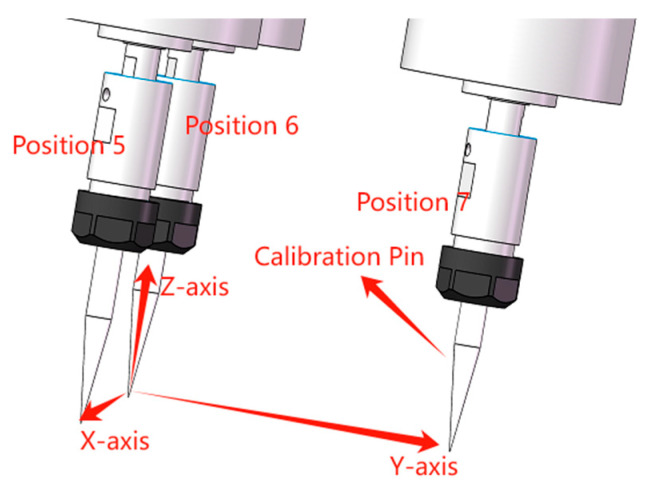
Schematic representation of posture calibration.

(4) Initial position adjustment and recording: Move the robot near the origin point, adjust the joints to align the tool coordinate system’s X and Z axes parallel to the corresponding axes of the base coordinate system, and then record the data parameters (for the 5th calibration point).

(5) Motion position recording: Maintaining the current orientation, move from Point 5 along the X and Y axes of the base coordinate system by specified distances, respectively, and then record the parameters for Points 6 and 7, as illustrated in Figure 4.

(6) Direction vector determination:

(16) PX=P6T2R2−P5T2R2

(17) PZ=P7T2R2−P5T2R2

(7) Transformation matrix computation: Orthonormalize PX, PY, and PZ, and substitute to obtain

(18) RT2E2=RE2R2−1⋅RT2R2

Combining the obtained *^E^*^2^*P_T_*_2_, we derive *^E^*^2^*T_T_*_2_:

(19) TT2E2=RT2E2PT2E201

**Figure 5:**
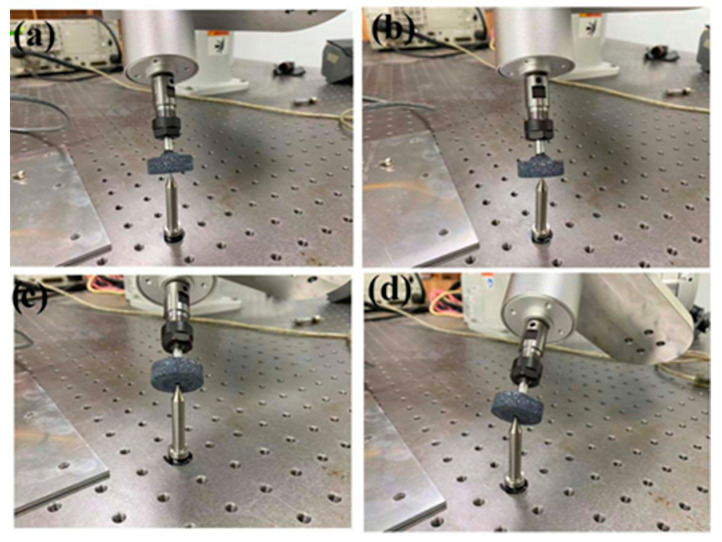
TCP calibration experimental diagram: (**a**) first pose, (**b**) second pose, (**c**) third pose, (**d**) fourth pose.

TCP Calibration Experiment:

By operating the Epson C4-A601S robot through its controller, the tool end-effector was repeatedly positioned at the same target point in four distinct spatial orientations (as illustrated in Figure 5). The end-effector pose data for each repetition were recorded. The experimental dataset is shown in Table 4.

The position matrix *^E^*^2^*P_T_*_2_ is obtained according to Equation (15):

(20) PT2E2=0.6442−129.4600−147.4710

Furthermore, since the *Y*-axis, *Z*-axis, and *X*-axis of the tool coordinate system are parallel to the *X*-axis, *Y*-axis, and *Z*-axis of the base coordinate system, respectively, PX and PY represent the directional vectors of the *Y*-axis and *Z*-axis in the tool coordinate system. The robot was controlled to move 50 mm along the *X*-axis and 50 mm along the *Y*-axis of the base coordinate system, with the data shown in Table 5.

The matrices *^R^*^2^*R_T_*_2_ and *^E^*^2^*T_T_*_2_ are computed using (18) and (19).

(21) RT2R2=010001100

(22) TT2E2=1000.6442010−129.4600001−147.47100001

## 3. Master Robot Modeling and Verification

### 3.1. Robot Coordinate Frame

**Figure 6:**
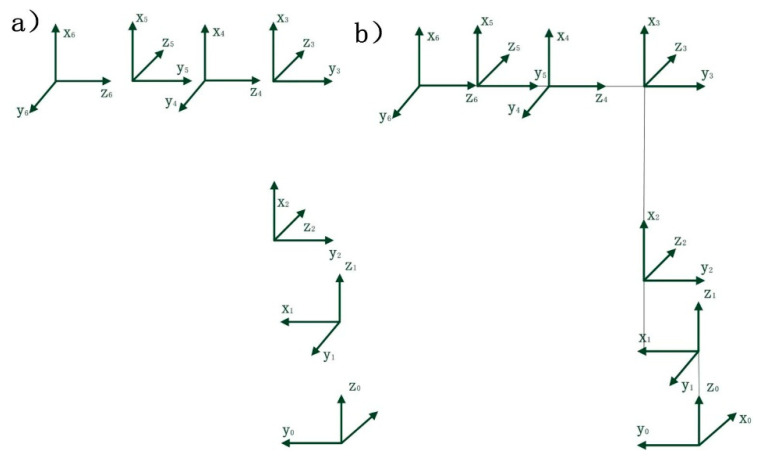
Link coordinate system diagram for robotics: (**a**) EPSONC4-A601S Robot, (**b**) EPSONC4-A901S Robot.

The EPSON C4-A901S robot is a serial articulated robot with six revolute joints. The modified Denavit–Hartenberg (D-H) parameter method was employed to establish the robot link coordinate system, as illustrated in Figure 6. Furthermore, the MDH parameters of the EPSON C4-A901S robot were obtained and are presented in Table 6. The EPSON C4-A601S robot differs in the MDH table only by the parameters a_3_ = 250 and d_4_ = −250.

*β* denotes the minor angular deflection between nominally parallel axes; *β_i_* represents the joint offset of the *i*-th joint; *θ_i_* is the angle between two adjacent links; *a_i_* indicates the link length; *α_i_* specifies the angle between two normals; and *d_ᵢ_* defines the distance between two normals.

### 3.2. Simulation and Verification of Robot MATLAB Model

**Figure 7:**
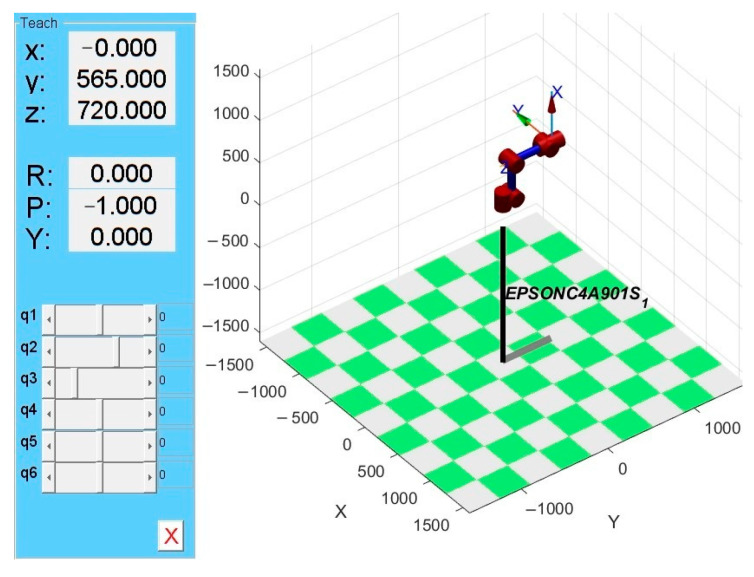
EPSONC4-A901S robot model.

Based on the MDH parameters presented in Table 5, the robotic model of EPSON C4-A901S was simulated using the Link function from MATLAB Robotics Toolbox. The simulation results of the EPSON C4-A901S robot are obtained as shown in Figure 7.

The homogeneous transformation matrix is obtained by solving the simulation model using the fkine command:

(23) $$T=\left[\begin{array}{cccc} 0 & -1 & 0 & 0\\ 0 & 0 & -1 & 565\\ 1 & 0 & 0 & 720\\ 0 & 0 & 0 & 1 \end{array}\right]$$

The homogeneous transformation matrix is obtained using the EPSON RC+ 7.0 software provided with the EPSON robot:

(24) $$T^{\prime }=\left[\begin{array}{cccc} 0 & -1 & 0 & 0\\ 0 & 0 & -1 & 565\\ 1 & 0 & 0 & 720\\ 0 & 0 & 0 & 1 \end{array}\right]$$

The MATLAB model demonstrates good agreement with the actual robot system.

**Figure 8:**
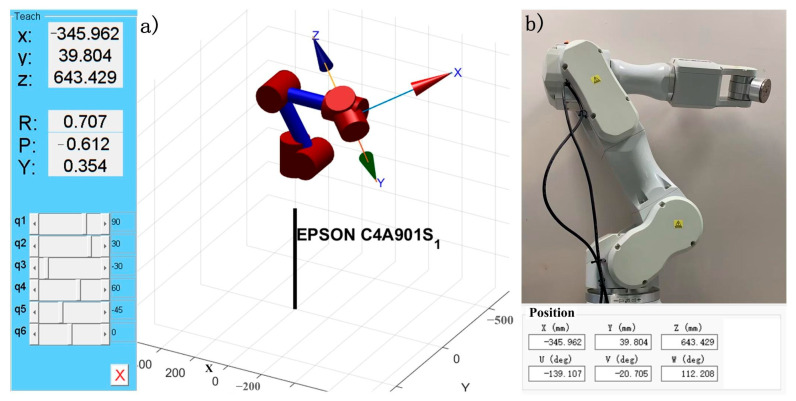
Position and orientation: (**a**) posture after simulated motion, (**b**) posture after physical movement.

Within the constrained workspace, Figure 8a shows the robot motion from initial position A (0, 0, 0, 0, 0, 0) to B (pi/2, pi/6, −pi/6, pi/3, −pi/4, 0) through teach pendant programming, while Figure 8b presents the results obtained via EPSON RC+ 7.0 software control.

**Figure 9:**
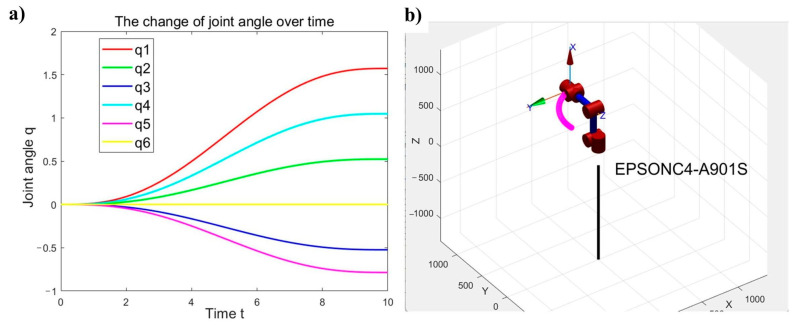
Joint and trajectory: (**a**) joint angle profiles of the robot, (**b**) end-effector trajectory of the robot.

By comparing the recorded position and orientation data of the robotic arm in Figure 8, it can be concluded that the MATLAB-based robot model parameters exhibit high consistency with the actual robotic system. As shown in Figure 9, the joint motion of the EPSON C4-A901S robot model demonstrates excellent smoothness over time without abrupt changes. This indicates that the established simulation model accurately characterizes the kinematic behavior of the physical system.

### 3.3. MATLAB-Based Modeling and Simulation of the Master Robot

For *^E^*^1^*T_T_*_1_, as illustrated in Figure 1, the gripper is connected to the end flange along the *Z*-axis via bolts, from which the transformation matrix T is derived through measurement.

(25) TT1E1=10000100001230.50001

Similarly, the transformation matrix *^T^*^1^*T_w_* is obtained.

(26) TwT1=10000100001100001

**Figure 10:**
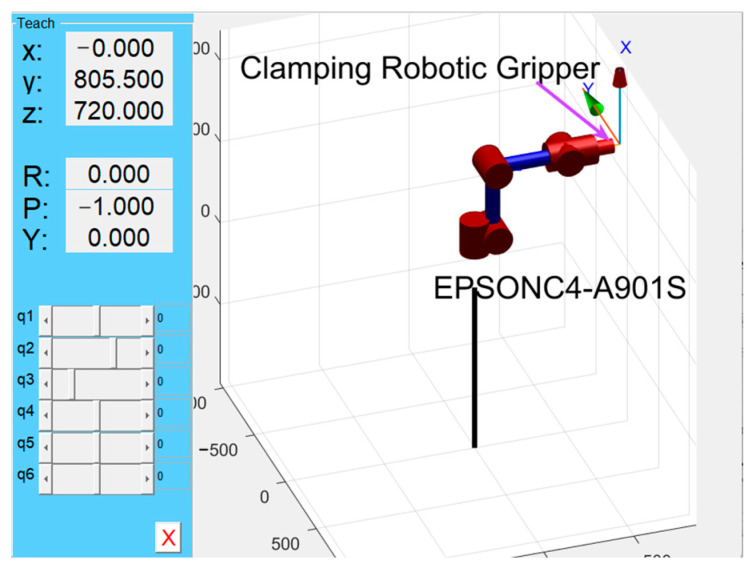
Master robot simulation model.

The master robot model was simulated using the tool function from the MATLAB Robotics Toolbox, as shown in Figure 10. In robotic kinematics and coordinate system definitions, the tool coordinate frame (TCF) is established as a local frame relative to the end-effector coordinate frame. Through the robot’s teach mode, the tool coordinate frame’s pose relative to the base frame is displayed, resulting in a change in the y-coordinate value from 565.0 to 805.5.

## 4. Collaborative Motion Simulation Analysis

A dual-robot grinding simulation platform was established using MATLAB Robotics Toolbox, with subsequent motion simulation analysis of the collaborative grinding system.

### 4.1. Establishment of the Dual-Robot Model

**Figure 11:**
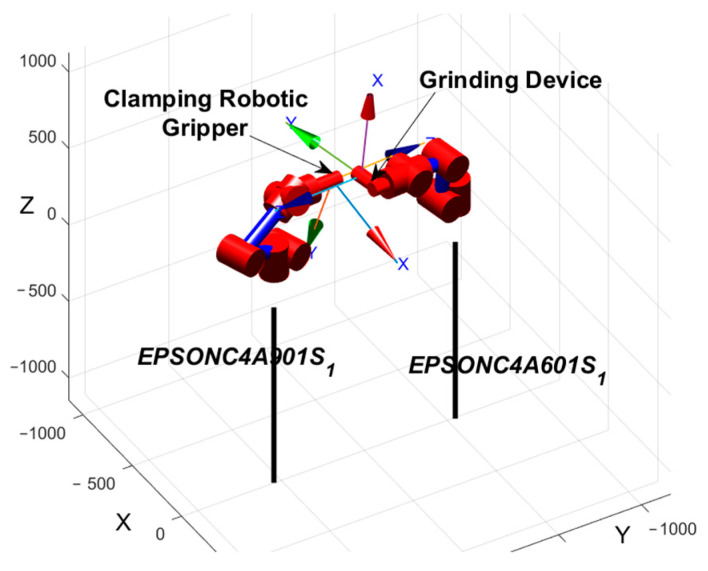
Dual-robot grinding simulation model.

Based on the derived *^R^*^1^*P_R_*_2_ and *^E^*^2^*T_T_*_2_ transformations, a dual-robot grinding simulation model was established using MATLAB Robotics Toolbox, as illustrated in Figure 11.

### 4.2. Cooperative Motion Simulation of Dual Robots

**Figure 12:**
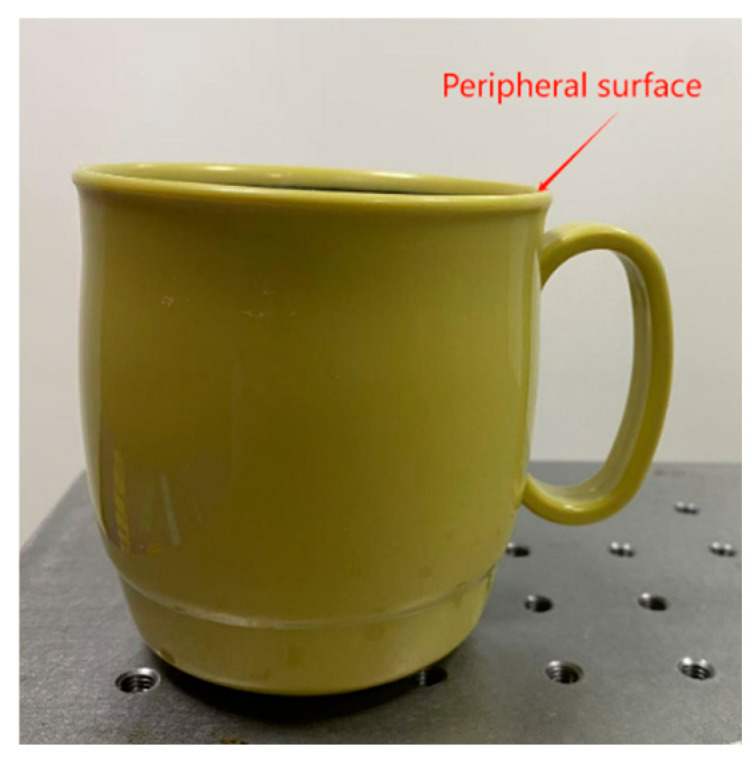
Workpiece.

Within constrained space, the cup’s edge surface is chosen for grinding; the actual workpiece is illustrated in Figure 12. Dual-arm trajectory control: The master robot performs linear spatial motion with fixed end-effector orientation, while the slave robot tracks a circular edge path on the cup within its restricted workspace.

The slave robot is positioned at the designated starting point for grinding, and its pose data is acquired to derive the transformation matrix of the workpiece relative to the base coordinate system as

(27) TwR1=0−0.01−1−343.22301−0.01−115.673100578.2340001

**Figure 13:**
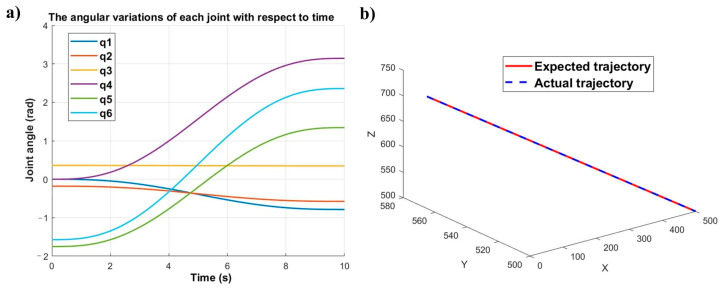
Joint and trajectory: (**a**) joint angle variation diagram of the master robot, (**b**) trajectory profile of the master robot.

**Figure 14:**
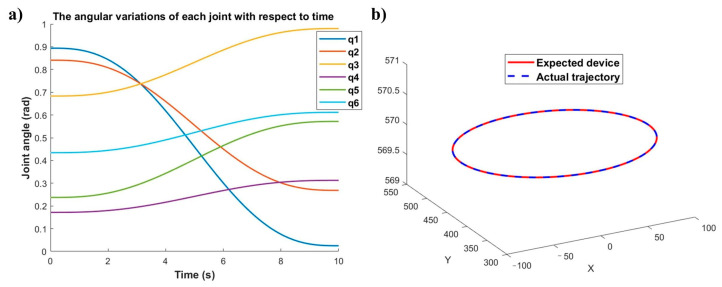
Joint and trajectory: (**a**) joint transformation diagram of the slave robot, (**b**) trajectory profile of the slave robot.

Based on the calibration results, Equations (1) and (2) are used to determine the end-effector trajectories and corresponding joint angles for both robotic arms. The simulation results obtained from these equations are shown in Figure 13 and Figure 14.

As shown in Figure 13a and Figure 14a, the joint angles of all six joints for both robots demonstrate smooth variation characteristics over time, with no observable abrupt joint motion phenomena. Furthermore, comparative analysis of Figure 13b and Figure 14b demonstrates satisfactory position tracking accuracy for both robotic manipulators. Thus, the feasibility and effectiveness of the proposed dual-robot grinding motion planning model are successfully validated.

## 5. Coordinated Motion Experiment

### 5.1. Optimal Single-Run Experimental Results of Dual-Robot Collaborative Motion

**Figure 15:**
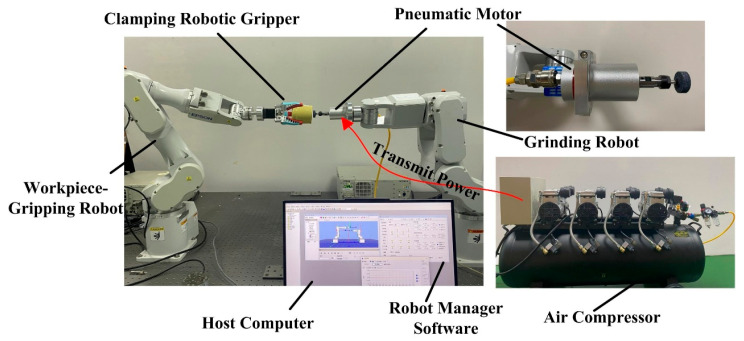
Dual-robot collaborative motion experimental platform.

As illustrated in Figure 15, the dual-robot coordinated motion hardware platform comprises the following components: two 6-DOF industrial robots (EPSON C4-A601s and EPSON C4-A901s), an upper computer, an air compressor, two RC700-A robot controllers, a pressure sensor, gripping tools, and grinding tools.

The system software layer constructs a master–slave dual-robot distributed control architecture through deep integration of EPSON RC+’s deterministic network configuration engine with SPEL+’s real-time communication instruction set. The architecture achieves reliable data transmission via the TCP/IP protocol stack, utilizing OpenNet and SetNet commands for network port parameter configuration and connection establishment, while enabling bidirectional real-time data interaction through ChkNet and Read instructions, thereby implementing a complete industrial communication management procedure of “parameter configuration → link establishment → data transmission → connection release”. During cooperative operation, the system performs real-time verification of robotic pose parameters using status monitoring commands such as LimZMargin in SPEL+ language. Upon detecting values exceeding preset safety thresholds, it instantly triggers hardware-level alarms and transmits emergency stop commands to the cooperating robot via TCP/IP protocol, achieving safety interlock protection in the dual-robot system.

The experimental procedure is as follows: First, establish a physical connection between the host computer and EPSON C4-A901S controller via Ethernet and USB interfaces, and then configure communication parameters to ensure link stability. Next, compile the master–slave robot motion programs in the SPE+ development environment with integrated TCP/IP communication modules for real-time status interaction. After compilation, download the programs to the controllers. The master robot plans motion trajectories containing collaborative waypoints and transmits them to the slave robot via a communication protocol, while both controllers synchronously convert trajectory commands into PWM signals to drive joint actuators. Simultaneously, motion state data and communication logs are acquired and stored using Call instructions, providing comprehensive datasets for subsequent analysis. Finally, execute dual-robot collaborative motion testing, repeating the experiment 10 times to statistically analyze trajectory tracking error stability metrics, followed by systematic evaluation to complete solution reliability verification.

**Figure 16:**
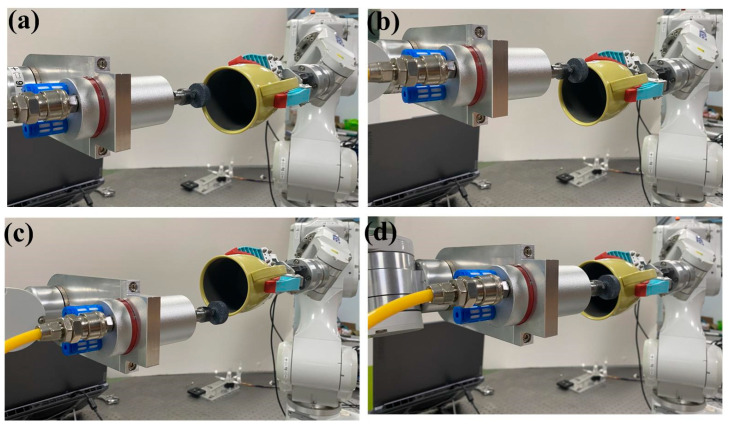
Key phases in dual-robot collaborative motion: (**a**) initial pose configuration, (**b**) first-quarter trajectory pose, (**c**) mid-trajectory pose, (**d**) third-quarter trajectory pose.

**Figure 17:**
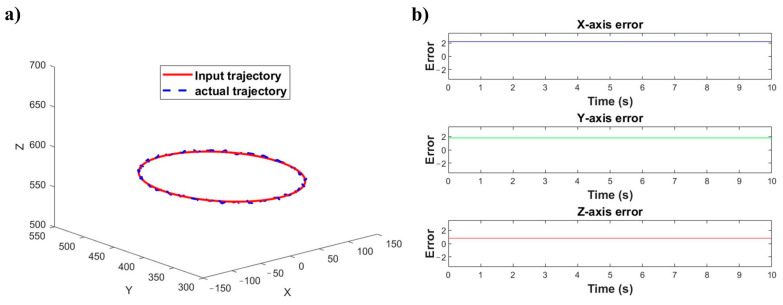
Trajectory and error: (**a**) slave robot motion tracking trajectory, (**b**) slave robot motion tracking error.

The critical aspects of dual-robot collaborative motion are illustrated in Figure 16. During dual-robot collaborative motion, the simulated reference trajectory and actual tracking trajectory of the master–slave robot end-effectors demonstrate high consistency, as shown in Figure 17a. This phenomenon preliminarily verifies that the dual-robot collaborative simulation model exhibits high accuracy and reliability in simulating real-world motion scenarios. To quantitatively evaluate the positional tracking accuracy of dual-robot collaborative operation, an in-depth analysis was conducted on the motion errors of the grinding tool along the X, Y, and Z axes (as shown in Figure 17b). Through precise measurement and data processing, the motion errors of the grinding tool along the X, Y, and Z axes were determined to be 2.158 mm, 1.81 mm, and 0.855 mm, respectively.

### 5.2. Repeatability Test Error Analysis

The XYZ-axis error data obtained from 10 repeated experiments are presented in Table 7.

The table displays error data and statistical analysis of the grinding tool along the X/Y/Z axes from 10 repeated experiments: The *X*-axis shows a mean error of 6.960 mm with a standard deviation of 3.007 mm, indicating significant systematic error and high dispersion characteristics in this axis. The *Y*-axis demonstrates a mean error of 4.230 mm with a 1.634 mm standard deviation and an error range of 1.810–7.678 mm, representing the most stable directional performance among the three axes. The *Z*-axis exhibits a mean error of 4.8129 mm with a 2.3148 mm standard deviation. While its mean value falls between the X and Y axes, it shows the highest coefficient of variation among all three axes. The experimental results demonstrate that the robot’s average trajectory tracking errors along the X, Y, and Z axes are all below 8 mm, meeting the accuracy requirements for medium- to low-precision tasks. These results demonstrate that the dual-robot collaborative simulation model can effectively guide practical operations, ensuring precise tracking of desired trajectories during coordinated motion while meeting stringent performance requirements for positional accuracy in collaborative tasks. This provides robust technical assurance for efficient and stable dual-robot collaborative operations.

### 5.3. Error Analysis Summary

The integrated method proposed in this study may exhibit errors due to the following three factors.

1. Mechanical structural errors: The robotic kinematic model relies on modified Denavit–Hartenberg (MDH) parameters (including link length, twist angle, joint offset, etc.). Deviations between the actual and theoretical parameters (e.g., machining tolerances or assembly errors) will induce kinematic calculation errors.

2. Vibration-induced errors: Pneumatic motors mounted on the robotic end-effector generate vibrations during motion, causing pose deviations, while vibrations in the robot’s internal sensors also introduce measurement errors.

3. Human-induced errors: Manual visual alignment of the calibration probe cannot achieve submillimeter-level tip positioning accuracy, resulting in calibration data deviations. Human-induced errors are the primary error source.

## 6. Conclusions

This study addresses the high-precision positioning challenge in dual-robot collaborative grinding and polishing by proposing an innovative, simple yet efficient calibration method that integrates the seven-point method with handshake calibration. Using a calibration needle as the reference tool, the method achieves rapid and precise positioning of dual robots, breaking through the accuracy bottleneck of system coordination in complex working scenarios.

This study establishes a system of kinematic constraint equations for dual robots based on their closed-loop motion chain configuration. Based on the process parameters and technical requirements of the grinding task, this study employs trajectory planning algorithms to generate precise grinding paths. Under the master–slave control architecture, this study first plans the ideal posture trajectory for the master robot, and then determines the slave robot’s pose parameters through inverse kinematics solving based on real-time updated collaborative motion constraint equations, thereby achieving efficient coordination of dual robots in grinding operations. During system calibration, the “handshake” calibration method is employed to align the base frames of dual robots. By establishing transformation relationships between the global coordinate system and individual robot coordinate systems, base frame installation errors are effectively eliminated. A seven-point calibration method is employed to achieve precise positioning of the Tool Center Point (TCP), where coordinated measurements via the robot’s end-effector ensure high-accuracy acquisition of TCP coordinates. The integration of these two calibration methods fulfills the high-precision operational requirements of dual-robot systems. In robotic modeling, an improved Denavit–Hartenberg (D-H) parameter method was employed to establish the kinematic model of the gripper robot (EPSON C4-A901S), with experimental validation confirming the model’s effectiveness and accuracy.

Based on the aforementioned calibration results, a dual-robot collaborative grinding simulation model was developed using the MATLAB Robotics Toolbox, followed by trajectory tracking experiments. The average error below 8 mm from 10 repeated experiments fully validates the accuracy and practical applicability of the integrated calibration method. This research significantly enhances the positioning accuracy of dual-robot systems and improves their adaptive recognition capability for end-effector tools, thereby establishing a robust yet straightforward technical solution for high-precision collaborative grinding of complex workpieces.

## Figures and Tables

**Table 1:** Comparison between the Handshake Method and AprilTag visual calibration.

	“Handshake” Method+ Seven-Point Calibration Method	AprilTag-Based Visual Calibration Method
Theoretical Foundation	Robotic Kinematic Equations and Coordinate Transformation Equation Sets	Vision-based Metrology and Image Processing
Data Source	Robotic Joint Angles and End-effector Kinematic Inverse Solutions	Camera Image Data and Marker Encoding Information
Hardware Cost	**Low-cost**	High-cost
Environmental Adaptability	Omnidirectional applicability	Marker Visibility Dependency
Application Fields	Traditional Industrial Robot Calibration	Wide-ranging Applications

**Table 2:** End-effector position of the master robot.

Serial Numbers	*X*-Axis Coordinate	*Y*-Axis Coordinate	*Z*-Axis Coordinate
1	−594.215	6.354	578.290
2	−544.215	6.154	577.740
3	−593.715	6.354	628.590
4	−593.915	−3.646	608.590

**Table 3:** End-effector position of the slave robot.

Serial Numbers	*X*-Axis Coordinate	*Y*-Axis Coordinate	*Z*-Axis Coordinate
1	485.858	0	572.732
2	535.857	0	572.732
3	485.858	0	622.732
4	485.858	−10	602.732

**Table 4:** Slave robot end-effector pose (identical spatial point).

Serial Numbers	1	2	3	4
X/mm	148.382	155.438	123.038	110.071
Y/mm	407.927	422.299	389.444	405.634
Z/mm	168.750	180.865	112.602	77.893
U/rad	75.788	67.484	90.894	97.282
V/rad	−31.891	−30.336	−40.751	−31.282
W/rad	177.146	−177.802	156.621	141.267

**Table 5:** From the end-effector pose of the slave robot (translation).

Serial Numbers	5	6	7
X/mm	0	50	0
Y/mm	415	415	465
Z/mm	570	570	570
U/rad	0	0	0
V/rad	−90	−90	−90
W/rad	−90	−90	−90

**Table 6:** MDH parameter table of EPSONC4-A901S robot.

Joint *i*	*α_i_*/rad	*a_i_*/mm	*θ_i_*/rad	*d_i_*/mm	*β_i_*/rad
1	0	0	pi/2	320	0
2	pi/2	100	pi/2	0	0
3	0	400	0	0	0
4	−pi/2	0	0	−400	0
5	pi/2	0	0	0	0
6	−pi/2	0	0	−65	0

**Table 7:** Statistical table of mean errors and standard deviations for X/Y/Z Axes in 10 experiments.

Serial Numbers	*X*-Axis	*Y*-Axis	*Z*-Axis
1	2.158	1.810	0.855
2	5.698	3.345	3.837
3	6.432	2.324	1.467
4	3.468	5.432	6.468
5	7.432	4.623	4.124
6	7.864	3.894	5.432
7	13.642	7.678	8.344
8	9.234	5.644	6.324
9	6.242	4.577	4.134
10	7.433	5.677	7.134
Mean Error	6.960	4.490	4.8129
Standard Deviation	3.007	1.743	2.3148

## Data Availability

The data used to support the findings of this study are available from the corresponding author upon reasonable request.
